# Genotype–phenotype correlations and clinical outcomes of genetic *TRPC6* podocytopathies

**DOI:** 10.1093/ndt/gfaf086

**Published:** 2025-05-19

**Authors:** Susan M McAnallen, Elhussein A E Elhassan, Sinead Stoneman, Filippo Pinto e Vairo, Marie C Hogan, Julia Hoefele, Michelle Clince, Poemlarp Mekraksakit, Silvia M Titan, Sofia Jorge, Joaquim Calado, Stéphane Decramer, Eloïse Colliou, Stéphanie Tellier, Telma Francisco, Aude Servais, Joséphine Cornet, Jonathan de Fallois, Claire Dossier, Roberta Fenoglio, Alessandra Renieri, Anna Maria Pinto, Sergio Daga, Lorenzo Loberti, Marc Fila, Luis F Quintana, Francesca Becherucci, Nathalie Godefroid, Astrid Dubrasquet, Tory Kálmán, Niamh Dolan, Bushra Al Alawi, Clodagh Sweeney, Michael Riordan, Maria Stack, Atif Awan, Ng Kar Hui, Hugh J McCarthy, Erik Biros, Trudie Harris, Kendrah Kidd, Stefanie Haeberle, Anthony J Bleyer, Andrew J Mallett, John A Sayer, Franz Schafer, Katherine A Benson, Emma McCann, Peter J Conlon

**Affiliations:** Department of Nephrology and Transplantation, Beaumont Hospital, Dublin, Ireland; Nephrology Department, St James's Hospital, Dublin, Ireland; Department of Medicine, Royal College of Surgeons in Ireland, Dublin, Ireland; Department of Nephrology and Transplantation, Beaumont Hospital, Dublin, Ireland; Department of Medicine, Royal College of Surgeons in Ireland, Dublin, Ireland; The European Rare Kidney Disease Reference Network (ERKNet), Heidelberg, Germany; Department of Nephrology and Transplantation, Beaumont Hospital, Dublin, Ireland; Division of Nephrology, Department of Medicine, University of British Columbia, Vancouver, British Columbia, Canada; Center for Individualized Medicine, Department of Clinical Genomics, Division of Nephrology and Hypertension, Mayo Clinic, Rochester, MN, USA; Division of Nephrology and Hypertension, Department of Medicine, Mayo Clinic, Rochester, MN, USA; Institute of Human Genetics, University Hospital, Ludwig-Maximilians University, Munich, Germany; Institute of Human Genetics, Klinikum rechts der Isar, Technical University of Munich, TUM School of Medicine and Health, Munich, Germany; Department of Nephrology and Transplantation, Beaumont Hospital, Dublin, Ireland; Division of Nephrology and Hypertension, Department of Medicine, Mayo Clinic, Rochester, MN, USA; Division of Nephrology and Hypertension, Department of Medicine, Mayo Clinic, Rochester, MN, USA; Department of Nephrology and Renal Transplantation of Hospital de Santa Maria, ULSSM, Lisbon, Portugal; NOVA Medical School, Faculdade de Ciências Médicas, Edifício CEDOC II, Rua Câmara Pestana, Lisbon, Portugal; Unidade de Nefrologia Pediátrica, Hospital de Dona Estefânia, Unidade local de Saúde de São José, Centro Clínico Académico de Lisboa, Rua Jacinta Marto, Lisbon, Portugal; Pediatric Nephrology and Internal Medecine. Pediatric Apheresis and Transplantation Children Hospital, Toulouse, France; Centre de Référence des Maladies Rénales Rares du Sud Ouest (SORARE) Membre du Réseau Européen ERKNet, Limoges University Hospital, Limoges, France; Département de Néphrologie et Transplantation d'Organes, Centre de Référence des Maladies Rénales Rares, Centre Hospitalier Universitaire de Toulouse, Toulouse, France; Pediatric Nephrology and Internal Medecine. Pediatric Apheresis and Transplantation Children Hospital, Toulouse, France; Centre de Référence des Maladies Rénales Rares du Sud Ouest (SORARE) Membre du Réseau Européen ERKNet, Limoges University Hospital, Limoges, France; Unidade de Nefrologia Pediátrica, Hospital de Dona Estefânia, Unidade local de Saúde de São José, Centro Clínico Académico de Lisboa, Rua Jacinta Marto, Lisbon, Portugal; Néphrologie et Transplantation rénale adulte, Hôpital Universitaire Necker Enfants Malades, APHP, Paris, France; Néphrologie et Transplantation rénale adulte, Hôpital Universitaire Necker Enfants Malades, APHP, Paris, France; Division of Nephrology, Department of Internal Medicine, University of Leipzig Medical Center, Leipzig, Germany; Department of Pediatric Nephrology, Robert-Debré Hospital, APHP, Paris, France; University Center of Excellence on Nephrological, Rheumatological and Rare Diseases Including Nephrology and Dialysis Unit and Center of Immuno-Rheumatology and Rare Diseases (CMID), San Giovanni Bosco Hub Hospital, ASL Città di Torino and Department of Clinical and Biological Sciences of the University of Turin, Turin, Italy; Medical Genetics, University of Siena, Siena, Italy; Med Biotech Hub and Competence Center, Department of Medical Biotechnologies, University of Siena, Siena, Italy; Genetica Medica, Azienda Ospedaliero-Universitaria Senese, Siena, Italy; Genetica Medica, Azienda Ospedaliero-Universitaria Senese, Siena, Italy; Medical Genetics, University of Siena, Siena, Italy; Med Biotech Hub and Competence Center, Department of Medical Biotechnologies, University of Siena, Siena, Italy; Medical Genetics, University of Siena, Siena, Italy; Med Biotech Hub and Competence Center, Department of Medical Biotechnologies, University of Siena, Siena, Italy; Genetica Medica, Azienda Ospedaliero-Universitaria Senese, Siena, Italy; Pediatric Nephrology Department, CHU Arnaud de Villeneuve – Montpellier University, Montpellier, France; Complex Glomerular Disease Unit (CSUR). Department of Nephrology and Kidney Transplantation, Hospital Clinic de Barcelona, University of Barcelona, IDIBAPS, Barcelona, Spain; Nephrology and Dialysis, Meyer Children's Hospital IRCCS, Florence, Italy; Department of Biomedical, Experimental and Clinical Sciences, University of Florence, Florence, Italy; Department of Pediatric Nephrology, Cliniques Universitaires Saint Luc, Brussels, Belgium; Centre de Référence des Maladies Rénales Rares du Sud Ouest (SORARE) Membre du Réseau Européen ERKNet, Limoges University Hospital, Limoges, France; Bordeaux University Hospital, Bordeaux, France; Pediatric Center, Semmelweis University, MTA Center of Excellence, Budapest, Hungary; Department of Paediatric Nephrology & Transplantation, Children's Health Ireland at Temple Street, Dublin, Ireland; Department of Paediatric Nephrology, Children's Health Ireland at Crumlin, Dublin, Ireland; Department of Paediatric Nephrology & Transplantation, Children's Health Ireland at Temple Street, Dublin, Ireland; Department of Paediatric Nephrology, Children's Health Ireland at Crumlin, Dublin, Ireland; Department of Paediatric Nephrology & Transplantation, Children's Health Ireland at Temple Street, Dublin, Ireland; Department of Paediatric Nephrology, Children's Health Ireland at Crumlin, Dublin, Ireland; Department of Paediatric Nephrology & Transplantation, Children's Health Ireland at Temple Street, Dublin, Ireland; Department of Paediatric Nephrology, Children's Health Ireland at Crumlin, Dublin, Ireland; Department of Paediatric Nephrology & Transplantation, Children's Health Ireland at Temple Street, Dublin, Ireland; Department of Paediatric Nephrology, Children's Health Ireland at Crumlin, Dublin, Ireland; Department of Paediatric Nephrology & Transplantation, Children's Health Ireland at Temple Street, Dublin, Ireland; Department of Paediatric Nephrology, Children's Health Ireland at Crumlin, Dublin, Ireland; Department of Paediatrics, School of Medicine & Medical Science, University College Dublin, Belfield, Dublin, Ireland; Paediatrics, Yong Loo Lin School of Medicine, National University of Singapore, Singapore; Department of Nephrology, Sydney Children's Hospital, Randwick, New South Wales, Australia; Nephrology Department, The Children's Hospital at Westmead, Westmead, New South Wales, Australia; College of Medicine and Dentistry, James Cook University, Queensland, Townsville, Australia; Department of Renal Medicine, Townsville University Hospital, Queensland, Townsville, Australia; Department of Renal Medicine, Townsville University Hospital, Queensland, Townsville, Australia; Section on Nephrology, Wake Forest University School of Medicine, Winston-Salem, NC, USA; The European Rare Kidney Disease Reference Network (ERKNet), Heidelberg, Germany; Heidelberg University Hospital, Center for Pediatric and Adolescent Medicine, Heidelberg, Germany; Section on Nephrology, Wake Forest University School of Medicine, Winston-Salem, NC, USA; College of Medicine and Dentistry, James Cook University, Queensland, Townsville, Australia; Department of Renal Medicine, Townsville University Hospital, Queensland, Townsville, Australia; Institute for Molecular Bioscience, The University of Queensland, Saint Lucia, Queensland, Brisbane, Australia; Renal Services, The Newcastle Hospitals NHS Foundation Trust, Newcastle upon Tyne, Tyne and Wear, UK; Biosciences Institute, Newcastle University, Newcastle upon Tyne, Tyne and Wear, UK; The European Rare Kidney Disease Reference Network (ERKNet), Heidelberg, Germany; Heidelberg University Hospital, Center for Pediatric and Adolescent Medicine, Heidelberg, Germany; School of Pharmacy and Biomolecular Sciences, Royal College of Surgeons, Dublin, Ireland; The Department of Clinical Genetics, Children's Health Ireland at Crumlin, Dublin, Ireland; Department of Nephrology and Transplantation, Beaumont Hospital, Dublin, Ireland; Department of Medicine, Royal College of Surgeons in Ireland, Dublin, Ireland; The European Rare Kidney Disease Reference Network (ERKNet), Heidelberg, Germany

**Keywords:** CKD, FSGS, podocytopathy, steroid-resistant nephrotic syndrome, *TRPC6*

## Abstract

**Background and hypothesis:**

Podocytopathy associated with likely pathogenic/pathogenic variants of Transient receptor potential cation channel subfamily C member 6 (*TRPC6*) (*TRPC6*-AP) has been recognized for about 20 years. As a result of its rarity however, the spectrum of clinical phenotypes and genotype–phenotype correlation of *TRPC6*-AP remains poorly understood. Here, we characterized clinical, histological and genetic correlates of familial and sporadic patients with *TRPC6*-AP.

**Methods:**

In this multicentre observational study, an online questionnaire followed by a systematic literature review was performed to create a cohort with comprehensive data on genetic and clinical outcomes [age of onset, clinical presentation, treatment response, kidney biopsy findings and progression to kidney failure (KF)]. Logistic regression, Cox proportional hazards model and Kaplan–Meier analyses investigated the associations between genetic variants and disease progression.

**Results:**

Among 87 families (96 familial and 45 sporadic cases), 31 distinct missense *TRPC6* variants (including 2 novel) were identified, with c.2683C>T p.(Arg895Cys) and c.523C>T p.(Arg175Trp) the commonest variants. Proteinuric kidney disease/nephrotic syndrome was the most common clinical presentation (83.7%), while focal segmental glomerulosclerosis was the most common histological finding (89.4%). By 33 (interquartile range 17–40) years, 48.9% (69/141) of patients had progressed to KF. Sporadic *TRPC6*-AP demonstrated an earlier progression to KF than familial cases (*P* = .001) and were more likely to present with nephrotic syndrome [odds ratio 4.34 (1.85–10.15); *P* = .001]. Gain-of-function *TRPC6* variants were more frequent in familial than sporadic *TRPC6*-AP (70.8% vs 44.4%; *P* = .004). Compared with patients with other *TRPC6* variants, patients with *TRPC6* p.R175W and p.R895C variants progressed to KF earlier [median kidney survival of 21 years, hazard ratio 2.985 (95% confidence interval 1.40–5.79); and 38 years, hazard ratio 1.65 (95% confidence interval 1.01–2.81), respectively, log-rank *P* = .005].

**Conclusion:**

Our study shows unique clinical and genetic correlations of *TRPC6*-AP, which may enable personalized care and promising novel therapies.

## Graphical Abstract

**Graphical Abstract:**
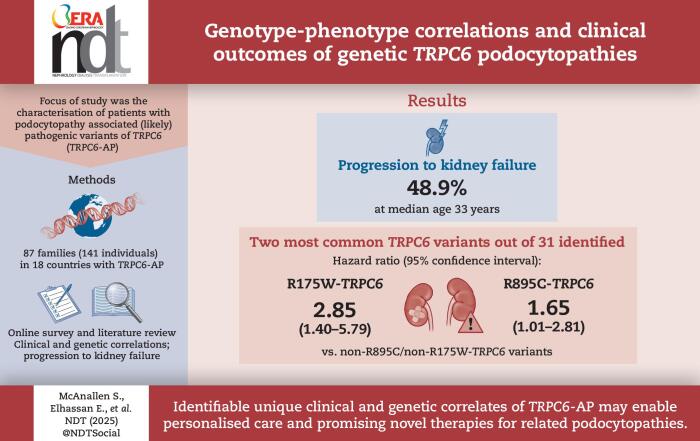

KEY LEARNING POINTS **What was known:**

• Heterozygous Transient receptor potential cation channel subfamily C member 6 (*TRPC6*) variants are associated with proteinuric kidney disorders (*TRPC6*-AP), often leading to kidney failure, with variable phenotype penetrance and disease progression.
• The dysfunctional calcium influx into the podocyte leads to focal segmental glomerulosclerosis, with gain-of-function (GOF-*TRPC6*) variants causing excessive calcium influx.
• Poor genotype–phenotype correlations have been demonstrated.

**This study adds:**

• Eighty-seven families (141 cases) with 31 disease-causing missense *TRPC6* variants, including 2 novel variants, were examined clinically and genetically.
• Sporadic *TRPC6*-AP were younger and present with nephrotic syndrome, while GOF-*TRPC6* variants were more common in familial patients.
• Patients with *TRPC6* p.(Arg175Trp) variant progressed earlier to kidney failure than other variants.

**Potential impact:**

• This investigation facilitates personalized care by allowing familial screening and informing kidney prognosis and treatment.
• This study highlights the importance of genetic diagnosis in reducing the burden of immunosuppression for individuals resistant to treatment.
• This study identifies families worldwide who may benefit from novel therapies targeting *TRPC6* currently under development.

## INTRODUCTION

Genetic testing has yielded significant insights into the physiology of the glomerular filtration barrier, facilitating the identification of likely pathogenic/pathogenic (LP/P) genetic variants causing dysfunction to the podocyte and its associated structural proteins in patients with steroid-resistant nephrotic syndrome (SRNS) or focal segmental glomerulosclerosis (FSGS), referred to as ‘podocytopathies’ [1–7]. Although patients with podocytopathies are often glucocorticoid-unresponsive and have limited options for targeted therapies [8, 9], establishing the underlying genetic basis of podocytopathies enables personalized management, allowing for immunosuppression-sparing approaches, prioritizing the protective effects of renin–angiotensin–aldosterone system inhibitors or using disease-specific therapies, such as coenzyme Q10 [9–11].

Transient receptor potential cation channel subfamily C member 6 (*TRPC6*; MIM #603 652) encodes a widely expressed protein in the podocyte that maintains its slit diaphragm integrity by mediating calcium entrance and cellular signalling that interacts with podocin and nephrin [12]. Variants causing podocytopathy-associated *TRPC6* (*TRPC6*-AP) have been linked to dominantly inherited monogenic FSGS [13, 14]. Functional studies that measure calcium influx into the podocyte demonstrate that gain-of-function (GOF-*TRPC6*) effect variants, often in the N-terminal ankyrin (ANK) repeat domain and C-terminal regions, increase calcium influx to the podocyte, precipitating cell damage and sclerosis [13–24]. Further studies showed that certain *TRPC6* missense variants substantially reduce calcium influx activity into the podocyte, mediating loss-of-function (LOF-*TRPC6*) effects and usually associated with an earlier onset of SRNS/FSGS in affected individuals [24]. Notably, variants in the same motif can express differential functional effects, such as p.(Arg895Cys), characterized as GOF-*TRPC6* [14], while p.(Arg895Lys) is LOF-*TRPC6* [24]. *TRPC6* protein-truncating variants have been described as having inactivating properties that completely disrupt calcium cellular entrance [25]. To date, genotype correlations have been shown to poorly influence disease progression [21].

In a recent multicentre case–control study, Wooden *et al.* utilized population-based next-generation sequencing data of 37 542 individuals to bridge the gap between the natural history and phenotypic features associated with *TRPC6*-AP [21]. They demonstrated a similar burden of *TRPC6* protein-truncating variants between cases and controls, suggesting a non-casual effect [21]. Furthermore, they described 39 families (64 cases) with 21 *TRPC6* missense variants, 11 of which are novel, clustering in regions other than the transmembrane domain of *TRPC6* [21]. Four variants exhibit the GOF-*TRPC6* effect using protein structural modelling [21]. Exploration of the clinical data and histopathological findings has revealed poor genotype–phenotype correlations [21]. Nonetheless, this paper has included missense *TRPC6* variants which are predicted to be benign/of uncertain significance, and further investigation is required.

To enhance our knowledge, we systematically reviewed 141 (114 published and 27 previously unreported) cases of podocytopathy secondary to LP/P *TRPC6* missense variants to decipher associated clinical, histological and genetic correlates.

## MATERIALS AND METHODS

### Study design and ethics

**Figure 1:**
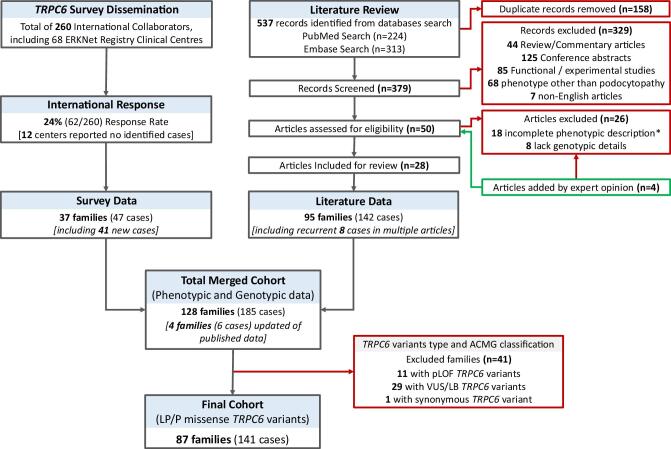
Flow diagram illustrating the survey distribution, literature review, exclusion criteria and final cohort of patients with LP/P non-synonymous *TRPC6* variants. ERKNet, the European Rare Kidney Disease Reference Network; LB, likely benign; *n*, number; VUS, variants of uncertain significance; pLOF, protein loss-of-function variants.

To understand the phenotypic and genetic outcomes of LP/P *TRPC6*-AP, an invitation e-mail was sent to 260 potential respondents, including authors, nephrologists and collaborators involved in inherited kidney disease (Fig. 1). All member centres associated with the European Rare Kidney Disease Reference Network were invited to contribute. Anonymized clinical, histological and genetic data related to affected individuals was obtained via an 82-item cross-sectional survey from 1 February 2023 until 30 June 2023 and structured into nine sections, with the following as primary areas: clinical presentation, diagnostic evaluation, management, disease progression and genetic diagnosis (Supplementary data, Table S1). We aimed to include all clinically and genetically affected individuals and their relatives with LP/P *TRPC6*-AP, proteinuria, SRNS and/or FSGS (see sections on genetic variants assessment and definitions). Thirty-seven initial families (47 cases) from centres in Europe, Australia, Singapore and North America were evaluated (Fig. 1).

Ethical approval was obtained from the Research Ethics Committee in Beaumont Hospital, Dublin (REC 19/28), and all centres participated voluntarily, following local legal and ethical guidelines.

### Search strategy and selection criteria

A systematic literature search was performed in MEDLINE (via PubMed) and Embase for published cases with *TRPC6*-AP since it was first published, 1 June 2005, to 30 December 2024. Utilizing the following terms ‘podocytopathy’, ‘FSGS’, ‘proteinuria’, ‘nephrotic syndrome’, ‘TRPC6’ and ‘Transient Receptor Potential Canonical 6’, 537 records were obtained. A complete search query is available in Supplementary data, Table S2. After excluding duplicates, non-original records (reviews, editorials and conference abstracts) and *in vitro* studies were excluded, along with articles with incomplete phenotypic or genetic details of *TRPC6* variants. Twenty-eight initial articles were identified and reviewed, yielding 95 families (142 cases), including information on 4 families (6 cases) updated from survey data (Fig. 1). Cases from the same cohort reported in multiple publications were identified and included once. Details of included and excluded articles are provided in Supplementary data, Tables S3–S5. The analysis yielded 128 families (185 cases) with phenotypic and genotypic data readily available for *TRPC6* variant classification by pooling data from the survey and literature review.

### Genetic variants assessment

Using the merged data, all *TRPC6* variants, identified using either exome sequencing or targeted gene panels related to monogenic nephropathies, were re-evaluated and classified by expert genetics researchers (E.A.E.E., F.P.V. and K.A.B.), according to the American College of Medical Genetics (ACMG) guidelines [26]. ACMG LP/P *TRPC6* variants were considered as ‘disease-causing’, based on previous reports, familial segregation and *de novo* data, *in silico* algorithms, and, when available, functional studies demonstrating *TRPC6* variant deleterious effects. Nucleotide positions were numbered in the coding sequence of human *TRPC6* mRNA (NM_004621.6) and referenced to the GRCh38/hg38 reference assembly. The pathogenicity of missense variants was predicted using Rare Exome Variant Ensemble Learner [27], Combined Annotation Dependent Depletion (version 1.7) [28] and AlphaMissense [29]. The minor allele frequency of *TRPC6* variants was retrieved from the Genome Aggregation Database (gnomAD v4.1.0, https://gnomad.broadinstitute.org/). Existing ClinVar database entries were checked (last accessed on 30 December 2024).

Following the report of Wooden *et al.* [21], protein LOF-*TRPC6* variants were excluded (*n* = 11 families). Variants that are likely benign or of uncertain significance with no evidence towards pathogenicity were excluded (*n* = 29 families). One survey case with a synonymous *TRPC6* NM_004621.6: c.75C>T (rs201051533) variant was excluded from the analysis, as the variant was classified as likely benign because it is relatively frequent and predicted to have no impact on the splice sequence using SpliceAI [30] and Pangolin [31], and classified as a likely benign/benign in the ClinVar database. Supplementary data, Table S6 provides all excluded *TRPC6* variants (*n* = 37) and Supplementary data, Table S7 summarizes clinical details of patients with *TRPC6* missense variants of uncertain significance. Eighty-seven families (141 cases) with LP/P *TRPC6*-AP comprised the final merged cohort.

### Data extraction, definitions, and disease endpoints

At initial presentation, age at disease onset, pattern of clinical manifestation, family history of SRNS/proteinuria and/or kidney disease, estimated glomerular filtration rate (eGFR) and proteinuria quantification were collected. Childhood-onset *TRPC6*-AP was defined by disease manifestation at age <18 years. Individuals with *de novo TRPC6* variants following parental genetic testing, or unaffected parents/relatives after a clinical evaluation, or no family history of kidney disease were considered sporadic *TRPC6*-AP, while familial *TRPC6*-AP were those with one or more clinically and/or genetically affected relatives. The follow-up duration is from the initial clinical presentation to the last follow-up.

Nephrotic syndrome was defined by urine protein:creatinine ratio (uPCR) >300 mg/mmol or equivalent, serum albumin <30 g/L and oedema, or as reported by the primary physician. Proteinuric chronic kidney disease was defined with a uPCR >15 mg/mmol ± eGFR <60 mL/min. Creatinine-based eGFR measurements were calculated utilizing the Chronic Kidney Disease Epidemiology Collaboration or modified Schwartz formula [32, 33], as applicable. If a native kidney biopsy was performed, histology was obtained, and the most prominent histopathological glomerular lesion was reported. Response to immunosuppressive treatment using corticosteroids and/or calcineurin inhibitors was evaluated against guidelines [34]. Post-transplant disease recurrence was defined as recurrence of nephrotic-range proteinuria (uPCR >300 mg/mmol) in patients who had undergone transplantation without an apparent alternative cause [35].

The primary outcome was the risk of kidney disease progression to kidney failure (KF) at last follow-up, defined by the need for kidney replacement therapy (chronic dialysis for ≥3 months or pre-emptive kidney transplantation). Also, we examined clinical and genetic determinants associated with other outcomes: initial presentation with SRNS/NS, sporadic *TRPC6*-AP and disease onset in childhood.

### Statistical analysis

All statistical analyses were performed using STATA SE software version 18.0 (Stata Corp., College Station, TX, USA). Descriptive data was employed with median and interquartile range (IQR) used for continuous variables, while count and frequency were used for the categorical variables. The Wilcoxon signed rank and Fisher exact tests were employed to compare the differences between groups in continuous and categorical variables, respectively. As applicable, logistic regression analyses calculated odds ratios (ORs) and confidence intervals (CIs) to assess the associations between variables. Kaplan–Meier methods and Cox regression analyses of time-dependent covariates were performed to predict progression to KF. Log-rank testing and hazard ratios (HRs) were employed to analyse significant differences and were accompanied by 95% CI. The incidence rate of KF and person-time was determined by dividing the number of failures by the person-time. A *P*-value of <.05 was considered significant.

## RESULTS

### Cohort characteristics

The analysed cohort comprised 87 families, including 45 sporadic cases and 96 affected individuals from 42 families. Twenty-seven cases were acquired from the online questionnaire and 114 from the literature review (Fig. 1). The median (IQR) age at presentation was 20.5 (8.4–31.5) years, with male and female patients almost equally represented (50.4% vs 48.2%). The majority of patients were Caucasian (58.9%), 19.1% were Asian and 2.1% were African. Clinical presentation varied across patients, predominantly presenting with proteinuric kidney disease (46.8%) and SRNS/NS (36.9%). Kidney biopsy was performed in 94/141 (66.7%), in which FSGS was the dominant histopathological diagnosis (89.4%). Over a median follow-up of 5 (IQR 1–11) years, 48.9% (69/141) progressed to KF at a median age of 33 (IQR 17–40) years. The median time from initial clinical presentation to KF was 4 (IQR 0.5–10) years, corresponding to an incidence rate of 16.8 (95% CI 13.2–21.3) per 1000 person-years (Supplementary data, Fig. S1). A summary of the phenotype characteristics and clinical outcomes per familial status is given in Table 1 and Supplementary data, Table S8 for full details.

**Table 1:** Clinical characteristics of patients with *TRPC6*-AP.

	**Families/cases**			
**Variables**	**Total (87/141)**	**Familial cases (42/96)**	**Sporadic cases (45 cases)** ^h^	***P*-** **value** ^i^
Gender, *n* (%)				
Male	71 (50.4)	50 (52.1)	21 (46.7)	.575
Female	68 (48.2)	44 (45.8)	24 (53.3)	
Unknown	2 (1.4)	2 (2.1)	0 (0)	
Ethnicity, *n* (%)				
Caucasian (White)	83 (58.9)	57 (59.4)	26 (57.8)	.151
East Asian (Asian)	27 (19.1)	20 (20.8)	7 (15.6)	
Hispanic	6 (4.3)	6 (6.2)	0 (0)	
African American (Black)	3 (2.1)	2 (2.1)	1 (2.2)	
Others^a^	22 (15.6)	11 (11.5)	11 (24.4)	
Age at initial presentation, years^b^	20.5 (8.4–31.5)	25 (15–35)	8.8 (5–25)	**<** **.001**
Age at last follow-up, years^b^	30 (17–42)	35 (21.5–46)	15 (8–34)	**<** **.001**
Duration of follow-up, years^b^	5 (1–11)	6 (1 -13)	3 (0.4–9)	.053
Clinical presentation, *n* (%)				
Proteinuric CKD	66 (46.8)	52 (54.2)	14 (31.1)	**<** **.001**
SRNS/NS	52 (36.9)	24 (25)	28 (62.2)	
Pregnancy-related presentation	12 (8.5)	9 (9.4)	3 (6.7)	
Asymptomatic at time of genetic testing	6 (4.3)	6 (6.2)	0 (0)	
Others	5 (3.5)	5 (5.2)	0 (0)	
Proteinuria at initial presentation, mmol/mg (*n* = 33)	300 (150–770)	300 (145–770)	459 (190–795)	.783
eGFR at initial presentation, mL/min (*n* = 37)	86.7 (33–99)	91 (20.4–104)	69 (60–91)	.771
Disease onset in childhood, *n* (%)^c^	58 (41.1)	28 (29.2)	30 (66.7)	**<** **.001**
Initial manifestation with KF, *n* (%)	21 (14.9)	15 (15.6)	6 (13.3)	1
Initial manifestation with SRNS/NS, *n* (%)	62 (44)	29 (30.2)	33 (73.3)	**<** **.001**
Kidney biopsy, *n* (%)^d^	94 (66.7%)	54 (57.9)	40 (90.9)	**<** **.001**
Histopathological findings, *n* (%)				
FSGS	84 (89.4)	50 (92.6)	34 (85)	.163
MCD	1 (1.1)	1 (1.8)	0 (0)	
Other histological patterns^e^	9 (9.5)	3 (5.6)	6 (15)	
Immunosuppression treatment, *n*/*N* (%)	22/60 (37.3)	10/40 (25)	12/20 (60)	**.011**
Response to immunosuppression treatment, *n* (%)^f^				
Complete or partial response	7 (30.4)	2 (20)	5 (38.5)	.405
Resistant to immunosuppression	16 (69.6)	8 (80)	8 (61.5)	
Progression of kidney disease, *n* (%)				
CKD	72 (51.1)	48 (50)	24 (53.3)	.722
KF	69 (48.9)	48 (50)	21 (46.7)	
Age at KF, years	33 (17–40)	35 (22.9–43.5)	11 (7–36)	**.001**
Time from presentation to KF, years	4 (0.5–10)	5 (1–11)	2 (0.4–9)	.103
Kidney transplantation, *n*/*N* (%) ^g^	42/50 (84)	30/36 (83.3)	12/14 (92.3)	1
Post-kidney Tx recurrence, *n* (%)	0 (0)	0 (0)	0 (0)	1

a Others comprise cases in which the self-reported ancestry is identified as Middle Eastern, Turkish, Iranian, Indian or mixed non-Caucasian.

b Missing data in five familial cases.

c Defined as disease-onset at age <18 years.

d Missing data in six cases out of a total of 132 kidney biopsies.

e Other histopathological patterns include C1q nephropathy (*n* = 1), immunoglobulin A (IgA) nephropathy and membranoproliferative glomerulonephritis (*n* = 1), IgA nephropathy and minor glomerular abnormality (*n* = 2), and diffuse mesangial sclerosis (*n* = 1).

f Response to immunosuppression was defined as per clinical published cases or the criteria applied from the online survey (see definitions in Materials and methods).

g Missing data in 19 cases. *n* indicates the number of cases who underwent kidney transplants, while *N* indicates the total number of cases with KF and available data on kidney transplant status. Seventeen cases were excluded as transplant status was not reported.

h Sporadic cases were defined as individuals who did not report any family history of kidney disease, had no clinically affected relatives after a clinical evaluation, or presented with a *de novo TRPC6* variant after parental genetic testing.

i Wilcoxon signed rank test was employed for continuous variables and Fisher exact test for categorical variables.

Continuous co-variants are presented as median (IQR).

CKD, chronic kidney disease; IS, immunosuppressive treatment; *n*, number of cases; *N*, total number of cases with available data; MCD, minimal change disease; Tx, transplantation.

### Clinical presentation and disease progression at last follow-up according to familial status

Next, we compared clinical findings between the familial and sporadic cases. Compared with familial cases, sporadic cases were younger at disease-onset and more likely to present with SRNS/NS (Table 1). Immunosuppression use was more common among sporadic *TRPC6*-AP at 60% (12/20) compared with familial cases at 25% (10/40) (*P* = .011), with no significant difference in treatment response. Additionally, sporadic cases exhibited earlier progression to KF than familial cases (Table 1). None of the 42 individuals with KF and known kidney transplant status experienced recurrence post-transplant, regardless of their family status. Significant differences were not observed in terms of gender, ethnicity and histological findings based on familial status.

### *TRPC6* variants distribution and functional characteristics

**Figure 2:**
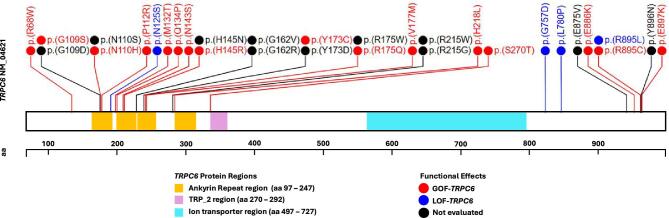
Distribution and functional effects of *TRPC6* variants. Lollipop plot illustrating the position of non-synonymous *TRPC6* variants in relation to a schematic representation of three protein regions: the ankyrin repeat region, transient receptor potential 2 region and ion transporter region (see key region below). Non-synonymous likely pathogenic/pathogenic *TRPC6* variants are annotated according to their functional impact on calcium channel function and are categorised into three groups: GOF (red solid circles), LOF (blue solid circles) and variants that were not functionally evaluated (black solid circles). The lollipop was created utilizing plot template of proteinpaint.stjude.org. aa, amino acid; TRP, transient receptor potential.

The distribution of LP/P missense variants in the coding sequence of *TRPC6* and their functional impact on calcium channel function is illustrated in Fig. 2. A total of 31 unique substitutions in *TRPC6*, including 2 previously unreported novel variants, were described (Table 2 and Supplementary data, Fig. S2). The two most prevalent *TRPC6* variants were c.2683C>T p.(Arg895Cys) (R895C-*TRPC6*) and c.523C>T p.(Arg175Trp) (R175W-*TRPC6*), identified in 18 and 13 families, respectively. Most variants were clustered in exon 2 (23 variants across 51 families) and exon 13 (5 variants across 29 families). *TRPC6* variants predominantly clustered in neighbouring regions that form an intracellular interface serving as an inhibitory domain of the TRPC6 channel, particularly the N-terminal ANK domain (17 variants in 41 families) and the C-terminal region (8 variants in 36 families), with none located within the transmembrane domain of TRPC6, as shown in Fig. 2. Sixteen variants were identified as exhibiting GOF-*TRPC6* effects, 4 exhibited LOF-*TRPC6*, while the other 11 *TRPC6* variants had not been functionally assessed previously (Supplementary data, Table S9). Except for the initial description of a large kindred by Winn *et al.* [36], no founder effect was identified. Geographical distribution of variants causing *TRPC6*-AP in this cohort is outlined in Supplementary data, Fig. S3.

**Table 2:** NS genetic variations in *TRPC6* (NM_004621.6) reported in this study.

			***In silico* prediction tools**					
**Inheritance** **^a^**	**Variant c.change; p.(change) (exon**)**^b^**	**Global AF****^c^** **(gnomAD)**	**CADD** **^d^**	**Alpha** **M** **issense**	**REVEL**	**Effects on Ca^2+^ channel function** **^e^**	**ACMG classification** (**evidence**)	**Families (*n***)
AD (also *de novo*)	c.2683C>T; p.(Arg895Cys) (Ex. 13)	0.000001859	32	0.986	0.91	GOF	Pathogenic (PS3, PM2_supp, PM5, PP3_mod)	18
AD	c.523C>T; p.(Arg175Trp) (Ex. 2)	0	32	0.826	0.55	NE	Pathogenic (PS4, PS2_mod, PM2_supp, PM5, PP1)	13
AD	c.643C>T; p.(Arg215Trp) (Ex. 2)	0.000003718	27.3	0.869	0.75	NE	Likely Pathogenic (PS4, PM2_supp, PM5, PP3)	5
AD	c.2689G>A; p.(Glu897Lys) (Ex. 13)	0	32	0.981	0.9	GOF	Pathogenic (PS4, PS3, PM2_supp, PP3_mod)	4
AD	c.2656G>A; p.(Glu886Lys) (Ex. 13)	0.00000062	31	0.989	0.85	GOF	Likely Pathogenic (PS4, PS3, PM2_supp, PP1, PP3_mod)	4
AD	c.335C>G; p.(Pro112Arg) (Ex. 2)	0	28.6	0.966	0.64	GOF	Pathogenic (PS4, PS3, PM2_supp, PM5, PS2_mod)	3
AD	c.374A>G; p.(Asn125Ser) (Ex. 2)	0.0003414	24.5	0.16	0.59	LOF	Likely Pathogenic (PS3, PP1, BS2)	3
AD	c.434A>G; p.(His145Arg) (Ex. 2)	0	23.9	0.979	0.29	GOF	Likely Pathogenic (PS4, PS3, PM2_supp, PP1)	3
AD	c.428A>G; p.(Asn143Ser) (Ex. 2)	0.00003841	24.9	0.206	0.46	GOF	Pathogenic (PS4, PS3, PM2_supp, PP1)	3
AD	c.2270G>A; p.(Gly757Asp) (Ex. 9)	0.0000006196	25.3	0.949	0.85	LOF	Pathogenic (PS4, PS3, PM2_supp, PP1, PP3_mod)	3
AD	c.2624A>T; p.(Glu875Val) (Ex. 12)	0	35	0.558	0.78	NE	Likely Pathogenic (PS4, PM2_supp, PP1, PP3_mod)	3
AD (also *de novo*)	c.326G>A; p.(Gly109Asp) (Ex. 2)	0	28.2	0.999	0.82	NE	Likely Pathogenic (PS2_mod, PM2_supp, PM5, PP1, PP3_mod)	2
AD	c.325G>A; p.(Gly109Ser) (Ex. 2)	0	28.2	0.983	0.78	GOF	Likely Pathogenic (PS3, PM2_supp, PM5, PP3)	2
AD	c.329A>G; p.(Asn110Ser) (Ex. 2)	0	25.7	0.44	0.47	NE	Likely Pathogenic (PS4, PM2_supp, PM5)	2
AD	c.524G>A; p.(Arg175Gln) (Ex. 2)	0.000004957	32	0.583	0.49	GOF	Likely Pathogenic (PS4, PS3, PM2_supp, PM5)	2
AD	c.2684G>T; p.(Arg895Leu) (Ex. 13)	0	32	0.992	0.87	LOF	Pathogenic (PS4, PS3, PS2_mod, PM2_supp, PM5, PP3_mod)	2
AD	c.202C>T; p.(Arg68Trp) (Ex. 2)	0.00002861	31	0.306	0.53	GOF	Likely Pathogenic (PS3, PM2_supp, PM5_supp, PP1)	1
AD	c.328A>C; p.(Asn110His) (Ex. 2)	0	26.5	0.728	0.51	GOF	Likely Pathogenic (PS3, PM2_supp, PM5_supp, PP1)	1
AD	c.395T>C; p.(Met132Thr) (Ex. 2)	0	25.5	0.97	0.7	GOF	Likely Pathogenic (PS3, PM2_supp, PP3)	1
AD	c.401A>C; p.(Gln134Pro) (Ex. 2)	0	27.2	0.996	0.7	GOF	Likely Pathogenic (PS3, PM2_supp, PP1, PP3)	1
AD	c.433C>A; p.(His145Asn) (Ex. 2)	0	26.8	0.912	0.37	NE	Likely Pathogenic (PM2_supp, PM5, PP1, PP3)	1
AD (also de novo)	c.484G>C; p.(Gly162Arg) (Ex. 2)	0	28.7	0.992	0.69	NE	Likely Pathogenic (PM2_supp, PM5_supp, PS2_mod, PP3_mod)	1
de Novo	c.485G>T; p.(Gly162Val) (Ex. 2)	0	28.4	0.987	0.73	NE	Likely Pathogenic (PS2_mod, PM2_supp, PP3_mod, PM5_supp)	1
de Novo	c.517T>G; p.(Tyr173Asp) (Ex. 2)	0	28.8	0.957	0.72	NE	Likely Pathogenic (PS2_mod, PM2_supp, PM5_supp, PP3_mod)	1
Singleton	c.518A>G; p.(Tyr173Cys) (Ex. 2)	0	28.2	0.699	0.76	GOF	Likely Pathogenic (PS2_mod, PM2_supp, PM5_supp, PP3_mod)	1
Singleton	c.529G>A; p.(Val177Met) (Ex. 2)	0.000001239	28.2	0.689	0.73	GOF	Likely Pathogenic (PS3, PM2_supp, PP3_mod)	1
AD	c.643C>G; p.(Arg215Gly) (Ex. 2)	0	28.2	0.958	0.71	NE	Likely Pathogenic (PS3, PM2_supp, PP3_mod)	1
AD	c.653A>T; p.(His218Leu) (Ex. 2)	0.000007436	22.6	0.204	0.57	GOF	Likely Pathogenic (PS3, PM2_supp)	1
AD	c.808T>A; p.(Ser270Thr) (Ex. 2)	0	25.8	0.933	0.9	GOF	Likely Pathogenic (PS3, PM2_supp, PP1, PP3_mod)	1
AD	c.2339T>C; p.(Leu780Pro) (Ex. 9)	0.000004957	27.4	0.567	0.68	LOF	Likely Pathogenic (PS3, PM2_supp, PP3)	1
AD	c.2686 T>A; p.(Tyr896Asn) (Ex. 13)	0	29.5	0.976	0.8	NE	Likely Pathogenic (PS4, PM2_supp, PP3_mod)	1

a Family history of parent–offspring inheritance is referred to as autosomal dominant ‘AD’. *De novo* indicates that parental genetic testing has confirmed their genetically unaffected state.

b Genomic alterations relative to Genome Reference Consortium Human Build 38.

c The Genome Aggregation Database version 4.1.0 accessed 30 December 2024.

d CADD score version 1.7.

e Functional effects of variant effect on calcium channel function were obtained from the following references [13–24].

AD, autosomal dominant; AF, allelic frequency; Ca^2+^, calcium; CADD, Combined Annotation Dependent Depletion; Ex., exon; gnomAD, the Genome Aggregation Database; GOF, gain of function of calcium channel function with increased calcium influx; LOF, loss of function of calcium channel function with decreased calcium influx; Mod, moderate; *n*, number of families; NE, not evaluated; REVEL, Rare Exome Variant Ensemble Learner Score; S, strong; Supp, supportive; VUS, variant of uncertain significance.

There were significantly higher GOF-*TRPC6* variants in familial cases than in sporadic cases (70.8% vs 44.4%; *P* = .004) (Table 3). No statistically significant differences were noted regarding the exonic distribution of *TRPC6* variants, the type of protein domains or the types of prevalent variants (all *P* ≥ .05).

**Table 3:** Genetic characteristics of patients with LP/P *TRCP6* variants.

	**Families/cases**			
**Variables**	**Total (87/141)**	**Familial cases (42/96)**	**Sporadic cases (45 cases)** ^a^	***P*-** **value** ^b^
Exonic distribution, *n* (%)				
Exon 2	83 (58.9)	56 (58.3)	27 (60)	.873
Exon 13	50 (35.5)	35 (36.5)	15 (33.3)	
Non-Exon 2/non-Exon 13	8 (5.6)	5 (5.2)	3 (6.7)	
Protein domains, *n* (%)				
N-terminal ANK domain region	65 (46.1)	42 (43.8)	23 (51.1)	.612
C-terminal region	58 (41.1)	40 (41.7)	18 (40)	
Non-N-terminal/non-C-terminal regions	18 (12.8)	14 (14.5)	4 (8.9)	
Functional effects of *TRPC6* variants on Ca^2+^ influx, *n* (%)				
GOF-*TRPC6*	88 (62.4)	68 (70.8)	20 (44.4)	**.004**
LOF-*TRPC6*	11 (7.8)	4 (4.2)	7 (15.6)	
not evaluated variants	42 (29.8)	24 (25)	18 (40)	
*TRPC6* variants prevalence, *n* (%)				
R895C-*TRPC6*	30 (21.3)	20 (20.8)	10 (22.2)	.261
R175W-*TRPC6*	19 (13.5)	10 (10.4)	9 (20)	
Non-R895C/non-R175W-*TRPC6*	92 (65.2)	66 (68.8)	26 (57.8)	

a Sporadic cases were defined as individuals who did not report any family history of kidney disease, had no clinically affected relatives after a clinical evaluation, or presented with a de novo *TRPC6* variant after parental genetic testing.

b Fisher exact test was employed for categorical variables.

Ca^2+^, calcium.

### Kidney survival and disease progression

**Figure 3:**
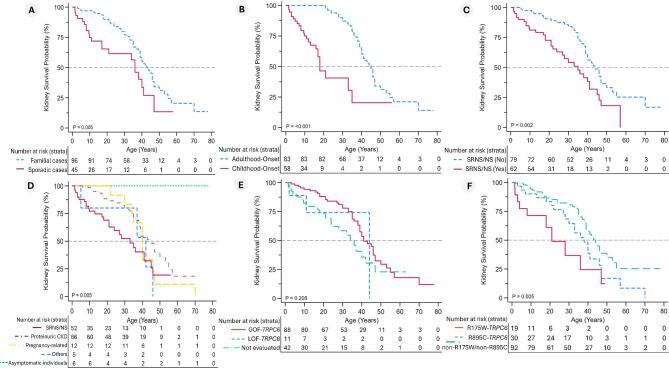
Kidney survival estimates of patients with LP/P non-synonymous *TRPC6* variants. The Kaplan–Meier survival curves show survival probability according to: (A) family history; (B) age at disease onset (adulthood vs childhood); (C) initial presentation with SRNS/NS; (D) pattern of clinical presentations; (E) functional impact on *TRPC6* variants on calcium channel function; and (F) *TRPC6* prevalent variants [c.523C>T p.(Arg175Trp) (R175W-TRPC6) variant, c.2683C>T p.(Arg895Cys) (R895C-TRPC6) compared with other *TRPC6* variants]. *P*-value from the log-rank test is given to compare the survival distributions among subgroups.

Kidney failure-free survival estimates of several phenotypic and *TRPC6* genotypic characteristics were examined (Fig. 3). Sixty-nine (48.9%) patients reached the primary kidney endpoint (KF—see definitions), with a median KF-free survival of 40 (95% CI 30–53) years for the entire cohort. Sporadic *TRPC6*-AP demonstrated an earlier progression to KF than familial cases (log-rank *P *= .005; Fig. 3A). Patients with disease-onset in childhood developed KF more frequently and exhibited accelerated progression to KF than those with adulthood-onset disease (log-rank *P* < .001; Fig. 3B). Compared with patients who presented with SRNS/NS at the onset, a more prolonged median kidney survival was associated with non-SRNS/NS presenters (log-rank *P* = .002; Fig. 3C) and presentations other than SRNS/NS (log-rank *P* = .005; Fig. 3D).

In terms of the most frequent *TRPC6* variants, patients with R175W-*TRPC6* and R895C-*TRPC6* variants progressed to KF earlier [Fig. 3F, HR 2.85 (95% CI 1.4–5.79) and HR 1.65 (95% CI 1.01–2.81), respectively] compared with patients with non-R175W/non-R895C-*TRPC6* variants (log-rank *P* = .005). In contrast, no significant kidney survival difference was identified based on exonic distribution, the type of protein domains, and the functional effects of *TRPC6* variants (all *P* ≥ .05) (Supplementary data, Fig. S4).

### Genomic and phenotypic correlations

Univariate and multivariable logistic regression analyses were performed to determine which clinical and genetic factors associated with *TRPC6*-AP were essential in the progression and severity of the disease. Regarding disease onset, in the univariate analysis, sporadic *TRPC6*-AP, the initial presentation with SRNS/NS and patients with LOF-*TRPC6* were associated with childhood-onset *TRPC6*-AP (Table 4A). In multivariate analysis, the risk of childhood-onset *TRPC6*-AP was six times higher in R175W-*TRPC6* patients compared with non-R895C/non-R175W-*TRPC6* variants (Table 4A).

**Table 4:** Logistic regression models outlining genetic associations with clinical presentation in childhood and sporadic *TRPC6*-AP.

	**Univariate analysis**		**Multivariate analysis**	
**Variables** **^a^**	**OR (95% CI)**	***P*-** **value**	**OR (95% CI)**	***P*-** **value**
**(A) Childhood presentation (<18 years)**				
Distribution of *TRPC6* variants				
Protein domains—ANK region (vs non-ANK/non-C-terminal regions)	2.52 (0.8–7.88)	.112	0.8 (0.16–3.97)	.786
Protein domains—C-terminal region (vs non-ANK/non-C-terminal regions)	1.47 (0.46–4.71)	.512	1.44 (0.39–5.24)	.576
Exonic distribution—exon 2 (vs other exons)	1.41 (0.71–2.82)	.321	0.35 (0.03–3.63)	.381
Exonic distribution—exon 13 (vs other exons)	0.55 (0.26–1.13)	.104	0.17 (0.02–1.91)	.154
Functional (effects on Ca^2+^ channel) status of *TRPC6* variant^b^				
LOF-*TRPC6* (vs GOF-*TRPC6*)	5.15 (1.27–20.87)	**.022**	1.16 (0.13–10.4)	.889
*TRPC6* variant prevalence^c^				
R895C-*TRPC6* (vs non-R895C/non-R175W-*TRPC6*)	0.98 (0.42–2.32)	.977	1.02 (0.2–5.11)	.979
R175W-*TRPC6* (vs non-R895C/non-R175W-*TRPC6*)	3.69 (1.28–10.62)	**.015**	6.69 (1.05–42.59)	**.044**
**(B) Sporadic *TRPC6*-associated podocytopathy**				
Distribution of *TRPC6* variants				
Protein domains—ANK region (vs non-ANK/non-C-terminal regions)	1.91 (0.56–6.5)	.297	1.79 (0.27–11.8)	.547
Protein domains—C-terminal region (vs non-ANK/non-C-terminal regions)	1.57 (0.45–5.45)	.474	1	1
Exonic distribution—exon 2 (vs other exons)	1.07 (0.52–2.2)	.851	1.99 (0.21–19.01)	.549
Exonic distribution—exon 13 (vs other exons)	0.87 (0.41–1.83)	.718	2.71 (0.32–22.95)	.361
Functional (effects on Ca^2+^ channel) status of *TRPC6* variant				
LOF-*TRPC6* (vs GOF-*TRPC6*)	5.95 (1.58–22.40)	**.008**	3.08 (0.82–34.8)	.985
*TRPC6* variant prevalence				
R895C-*TRPC6* (vs non-R895C/non-R175W-*TRPC6*)	1.21 (0.52–3.07)	.597	3.12 (0.73–13.31)	.124
R175W-*TRPC6* (vs non-R895C/non-R175W-*TRPC6*)	2.28 (0.83–6.26)	.108	0.80 (0.19–3.44)	.773

a The following clinical co-variates were adjusted to genetic factors in the multivariate model: sex, familial status (familial vs sporadic), age of initial presentation (years), initial presentation with SRNS/NS and ethnicity. To avoid the collinearity, models of childhood presentation and sporadic disease were only depended on familial status and age of initial presentation, respectively.

b Forty-five individuals with *TRPC6* variants that were ‘not evaluated’ functionally were excluded from the univariate analysis.

c Comparing the two most prevalent *TRPC6* variants—p.(Arg895Cys) (R895C-*TRPC6*) and p.(Arg175Trp) (R175W-*TRPC6*)—vs the remaining variants (non-R895C/non-R175W-*TRPC6*).

d Regarding disease onset, in the univariate analysis, sporadic *TRPC6*-AP [OR 4.86 (2.27–10.38); *P* < .001], the initial presentation with SRNS/NS [OR 3.59 (1.77–7.25); *P* < .001] and patients with LOF-*TRPC6* [OR 5.15 (1.27–20.87); *P* = .022] were associated with childhood-onset of *TRPC6*-AP.

e Regarding familial status, in univariate analysis, sporadic *TRPC6*-AP were more likely to present with SRNS/NS [OR 6.35 (2.88–14.01); *P* < .001], and harbour LOF-*TRPC6* variants [OR 5.95 (1.58–22.4); *P* = .008]. Each year of age upon presentation decreased the risk of sporadic *TRPC6*-AP by 7% [OR 0.93 (0.91–0.96); *P* < .001]. SRNS/NS at initial presentation was the only clinical co-variate in multivariate analysis that predicted sporadic *TRPC6*-AP [OR 4.34 (1.85–10.15); *P* = .001].

Ca^2+^, calcium; GOF, gain of function of calcium channel function with increased calcium influx; LOF, loss of function of calcium channel function with decreased calcium influx.

Regarding familial status, sporadic *TRPC6*-AP were more likely to present with SRNS/NS and to harbour LOF-*TRPC6* variants, whereas in the multivariate analysis, none of the genetic covariates were statistically significant (Table 4B).

In the multivariate Cox hazard model, none of the genetic covariates predicted progression to KF except age at initial presentation in individuals with *TRPC6*-AP [HR 0.89 (0.86–0.93); *P* < .001] (Table 5).

**Table 5:** Time-dependent Cox proportional hazards regression models of genetic determinants associated with the development of KF in LP/P *TRPC6*-AP.

**Variables** **^a^**	**Median age of kidney survival (95% CI), years**	**Univariate analysis**, **HR (95% CI)**	***P* *-*** **v** **alue**	**Multivariate analysis**, **HR (95% CI)**	***P*-** **value**
Distribution of *TRPC6* variants					
*TRPC6* protein domains—ANK region (vs non-ANK/non-C-terminal regions)	41 (28–50) vs 40 (35–NE)	1.82 (0.78–4.21)	.161	1.82 (0.52–6.36)	.350
*TRPC6* protein domains—C-terminal region (vs non-ANK/non-C-terminal regions)	40 (27–47) vs 40 (35–NE)	1.74 (0.77–3.96)	.181	1	1
Exonic distribution—exon 2 (vs other exons)	40 (30–55) vs 40 (28–47)	0.89 (0.56–1.43)	.643	1.62 (0.26–10.04)	.602
Exonic distribution—exon 13 (vs other exons)	40 (28–47) vs 40 (28–53)	1.03 (0.64–1.67)	.875	2.29 (0.35–14.82)	.385
Functional (effects on Ca^2+^ channel) status of *TRPC6* variant					
LOF-*TRPC6* (vs GOF-*TRPC6*)	44 (11–44) vs 41 (33–55)	1.54 (0.47–5.04)	.475	1.72 (0.31–9.53)	.530
*TRPC6* variant prevalence					
R895C-*TRPC6* (vs non-R895C/non-R175W-*TRPC6*)	38 (27–47) vs 44 (35–NE)	1.65 (1.01–2.81)	**.045**	0.61 (0.20–1.77)	.360
R175W-*TRPC6* (vs non-R895C/non-R175W-*TRPC6*)	21 (8–36) vs 44 (35–NE)	2.85 (1.40–5.79)	**.004**	1.39 (0.41–4.67)	.590

^a^The following as co-variates were adjusted to genetic factors in the multivariate model to investigate the association between predictor variables and the kidney survival time: sex, initial presentation with SRNS/NS (yes vs no), presentation in childhood (yes vs no), ethnicity (Caucasian vs non-Caucasians), familial status (familial vs sporadic) and age of initial presentation. In univariate Cox proportional hazard model, patients with childhood-onset *TRPC6*-AP [HR 4.61 (2.67–7.96); *P* < .001], sporadic *TRPC6*-AP [HR 2.04 (1.21–3.45); *P* = .007] and presenting with SRNS/NS [HR 2.06 (1.27–3.34); *P* = .003] were more likely to progress to KF. However, in the multivariate analysis none of the genetic factors was statistically significant, the age at initial presentation was the most accurate determinant of progression to KF in individuals with *TRPC6*-AP [HR 0.88 (0.85–0.93); *P* < .001].

Ca^2+^, calcium; CKD, chronic kidney disease; GOF, gain of function of calcium channel function with increased calcium influx; LOF, loss of function of calcium channel function with decreased calcium influx; NA, not applicable; NE, not estimable.

## DISCUSSION

Genotype–phenotype correlations enable the identification of critical observations regarding the pathophysiology of the affected gene and its impact on clinical outcomes. Here, we comprehensively examined the largest cohort of a rare podocytopathy caused by LP/P *TRPC6* variants. We have gathered data of 141 cases (84 families) from major genomic medicine centres and reviewed published literature. We have identified several essential attributes of *TRPC6*-AP, such as its global distribution, the influence of various specific variants on kidney phenotype, including age of KF, and the distinction in phenotypes between sporadic and familial *TRCP6*-AP. We demonstrate that patients with *TRPC6*-AP exhibit heterogeneous clinical manifestations, typically presenting with nephrotic syndrome or subnephrotic-range proteinuria, indicating variation in the severity and type of symptoms despite the same underlying genetic cause. Additionally, a small percentage of familial cases displayed incomplete phenotypic penetrance (4.3%). Due to its prominent tissue expression, the TRPC6 channel is involved in numerous cellular functions, mainly enabling the cellular entrance of calcium ions, and interacts with glomerular cytoskeleton proteins [37, 38]. As a result, *TRPC6* plays a role in a diverse array of genetic and non-genetic diseases [38]. In *TRPC6*-AP, this phenotypic variability is characteristic of different expressivity. It highlights how genetic variants can lead to a spectrum of clinical presentations, influenced by factors such as modifier genes, environmental influences or other regulatory mechanisms [38, 39].

*TRPC6-*AP also has an aggressive course, with nearly half of patients progressing to KF in young adulthood. Family sizes in our series were typically smaller than those in the initial description [36]. The prognosis was worse for sporadic cases, which were more likely to manifest in childhood and with nephrotic syndrome. Moreover, patients with the most prevalent variants in this cohort, R175W-*TRPC6* and R895-*TRPC6*, progressed to KF earlier compared with individuals with other variants. Disease did not recur post transplant, which is indicative of the healthy donor kidney's normal TRPC6 expression *in situ* and may indicate that the disease is not immunogenic, in contrast to primary FSGS [40].

A recent multicentre study has characterized the landscape of podocytopathy caused by missense *TRPC6* variants [21]. Our results corroborate and expand upon their findings in multiple areas. Firstly, *TRPC6*-AP results in a variable disease presentation and progression to KF, regardless of sex and affected TRPC6 protein domains. All variants were predicted to be clustered across the encoded protein but not within the six transmembrane domains, with no particular variant hotspot region identified. Secondly, the two cohorts had a comparable period from clinical diagnosis to KF, 4 years versus 5 years. Thirdly, familial individuals displayed noticeable disease variability, characterized by different phenotypic expressivity, and family sizes were distinctively small. However, notably, Wooden *et al.* did not identify a difference in progression to KF between the two most prevalent *TRPC6* variants identified in their cohort, R895C-*TRPC6* and E897K-*TRPC6*—both have GOF-*TRPC6* effects. Our larger cohort identified statistically significant rank-like differences in kidney survival between individuals with R175W-*TRPC6* and R895C-*TRPC6* compared with the other variants. The R175W-*TRPC6* variant, which has not been evaluated functionally so far, has been reported in 13 families (19 cases) who, on average, reached KF at a median age of 21 years, compared with 38 and 44 years to R895C-*TRPC6* and non-R175W/non-R895C-*TRPC6* variants, respectively. This finding suggests that R175W-*TRPC6* variant might exhibit loss of calcium ion influx into the podocyte *TRPC6*, leading to earlier progression to KF.

The strengths of this study are several. The collaborative distribution of our survey and the incorporation of a comprehensive literature review of previously published cases improved the characterization of *TRPC6*-AP disease evolution. Only LP/P variants of *TRPC6*-AP were included, representing patients from 18 countries worldwide to ensure diversity and inclusivity of the largest population to date. To demonstrate its translational impact on patients with *TRPC6*-AP, we studied a series of clinical endpoints relevant to daily practice, encompassing progression to KF and phenotypic traits, which revealed novel indicators suggesting *TRPC6* variant-specific characteristics on disease progression. Yet, several limitations should be noted. The retrospective design may introduce inherent biases, such as incomplete or missing data and the selection of severely impacted individuals. Detailed characteristics of meaningful phenotypic features at baseline, such as eGFR decline, proteinuria quantification and histological details, remain lacking. Additionally, the potential ramifications of specific *TRPC6* variants, notably excluded some missense variants of uncertain significance, are constrained by the dependence on ACMG-based classification, which may potentially over- or under-call these variants. Although we have used all available evidence to apply the selection criteria of submitted variants, the pathogenicity remains uncertain for some variants, particularly in light of the lack of functional studies or additional affected probands. This offers a promising opportunity to functionally characterize these variants and better clarify their cellular effects.

Overall, our study advances the understanding of genotype–phenotype correlations of podocytopathy caused by LP/P missense *TRPC6* variants. Future research endeavors are anticipated to examine potential targeted therapies for patients with this rare disease.

## Supplementary Material

gfaf086_Supplemental_Files

## Data Availability

The data underlying this article will be shared on reasonable request to the corresponding and named senior author.
