# Supplementary material for: Genotype–phenotype correlations and clinical outcomes of genetic TRPC6 podocytopathies
Source: Nephrol Dial Transplant. 2025 May 19;41(1):79–91. doi: 10.1093/ndt/gfaf086 (PMC12836101; doi:10.1093/ndt/gfaf086)
Supplement: gfaf086_Supplemental_Files [file gfaf086_Supplemental_Files.zip › Supplementary Materials_NDT_TRPC6_R2_01303_2024-crx.pdf]

Supplementary Materials

Supplementary information is available at NDT's website.

Supplementary Figures

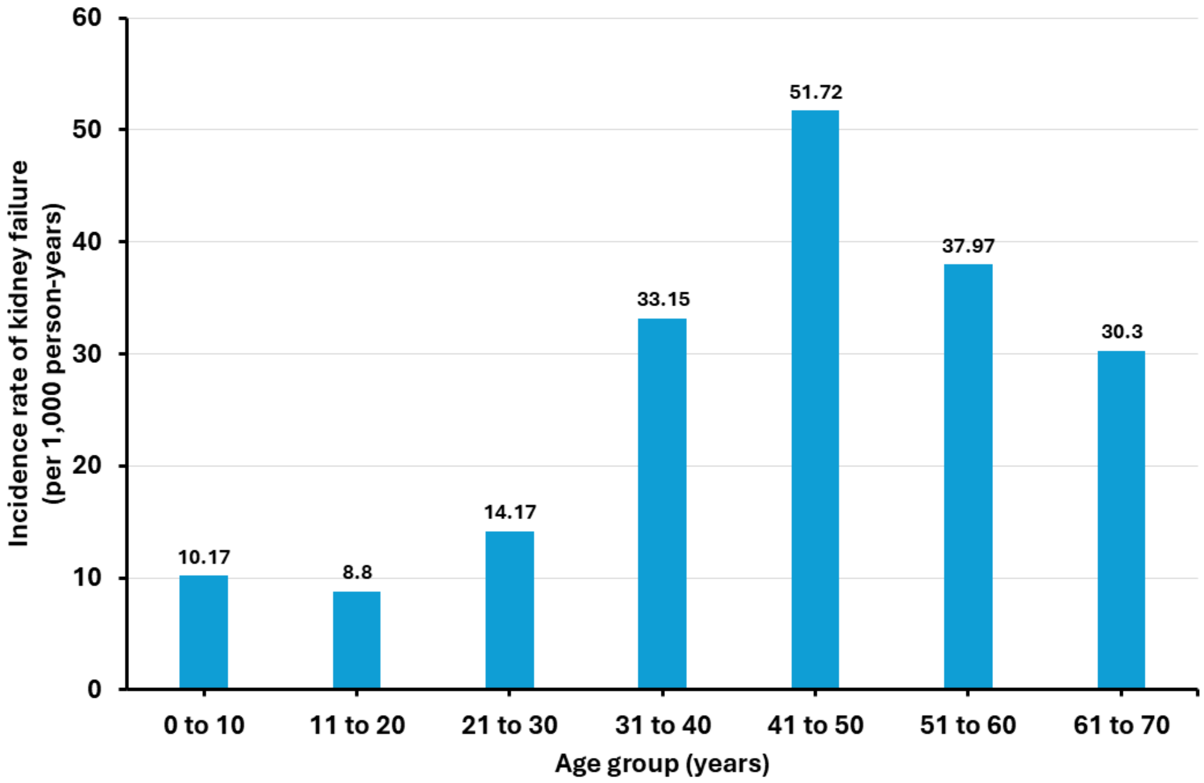

Figure S1 | Incidence rate of kidney failure (per 1,000 person-years) in patients with Pathogenic/Likely Pathogenic *TRCP6* variants.

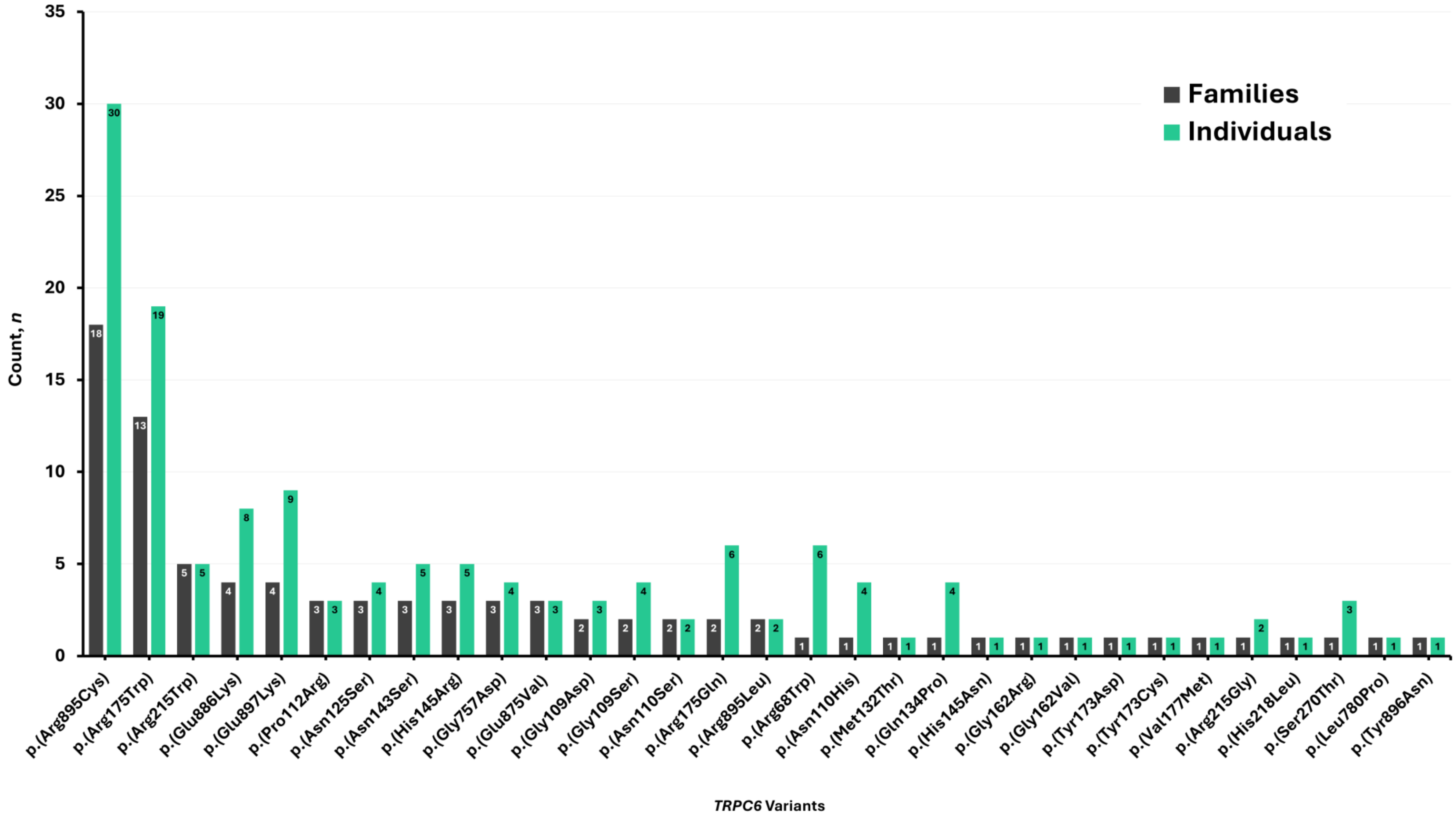

Figure S2 | *TRPC6* Variants Distribution based on the number of the affected families and individuals

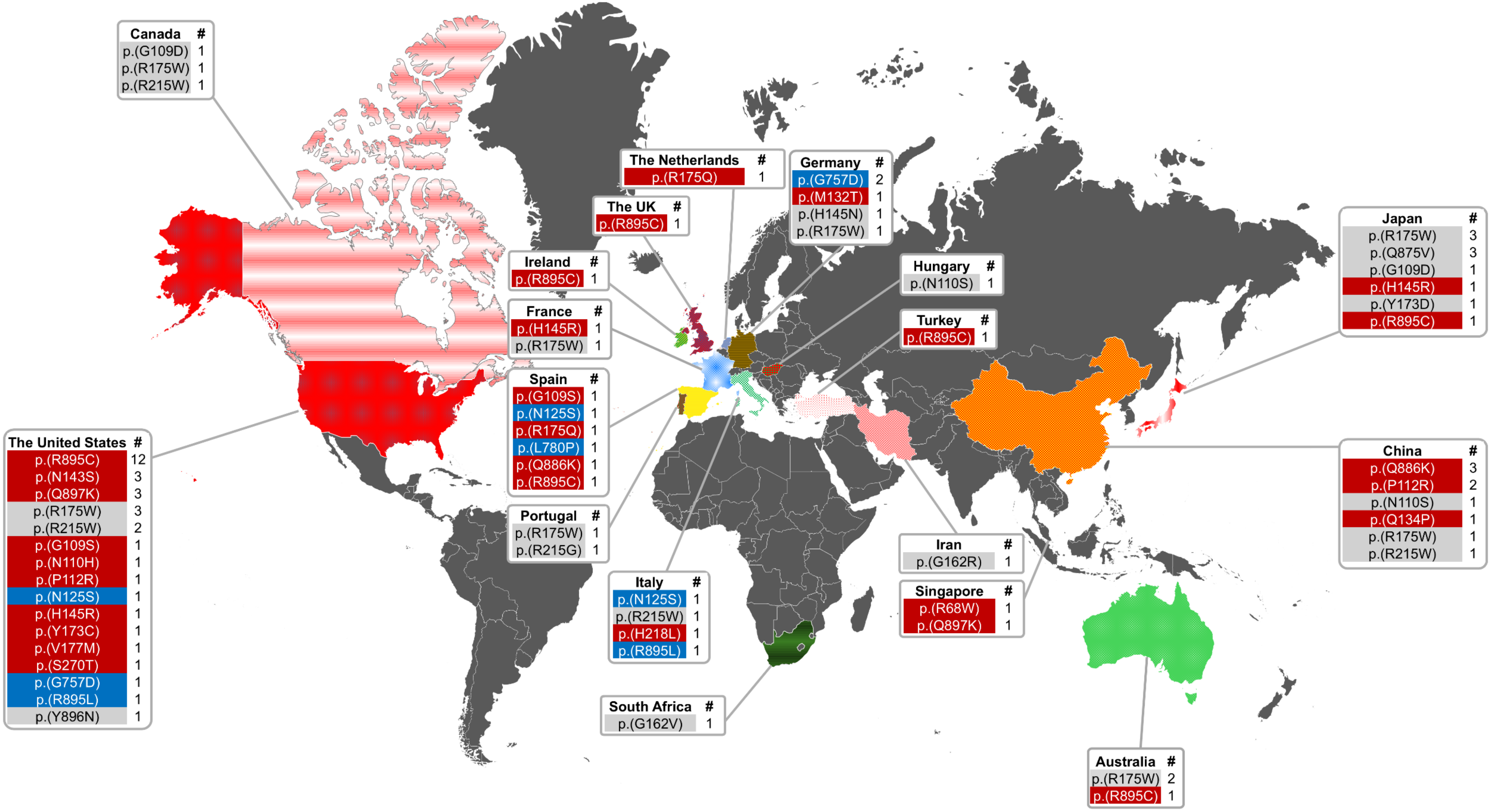

Figure S3 | Geographical Distribution of Likely Pathogenic and Pathogenic variants causing *TRPC6*-Associated Podocytopathy

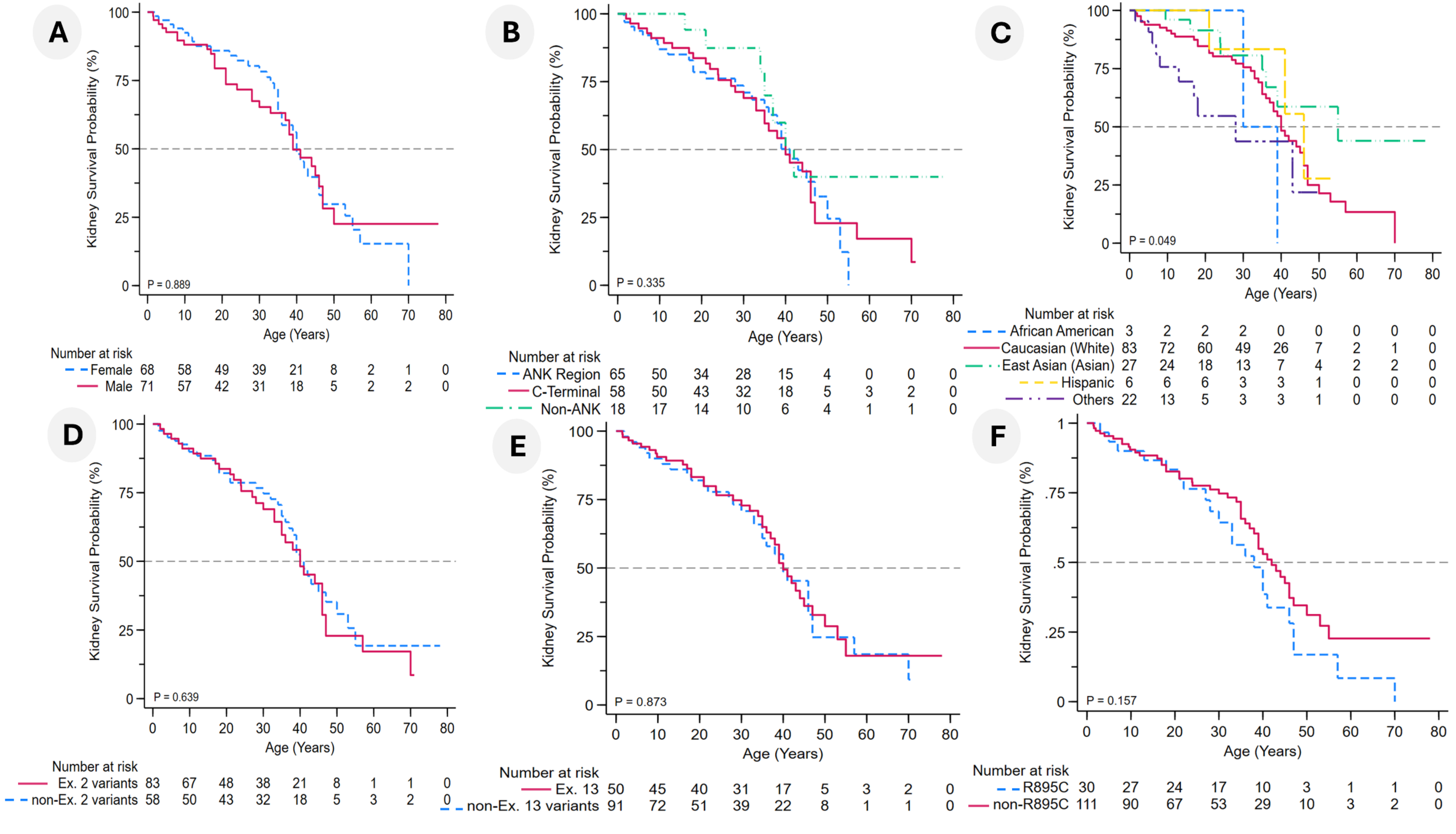

Figure S4 | Kaplan–Meier Kidney Survival Estimates
